# Increasing Beef Production in the Northern Region of the Republic of Kazakhstan Using the Genetic Resources of Aberdeen Angus Cattle of Different Genotypes

**DOI:** 10.3390/ani14243584

**Published:** 2024-12-12

**Authors:** Pavel Shevchenko, Bakhit Baimenov, Vadim Ulyanov, Zhanaidar Bermukhametov, Kulyay Suleimanova, Jan Miciński, Raushan Rychshanova, Inna Brel-Kisseleva

**Affiliations:** 1 Research and Innovation Center, Research Institute of Applied Biotechnology, NLC «Akhmet Baitursynuly Kostanay Regional University», Kostanay 110000, Kazakhstan; pavel87011339688@gmail.com (P.S.); bahytbajmenov@gmail.com (B.B.); zhanaidar007@gmail.com (Z.B.); rrychshanova@gmail.com (R.R.); 2 Testing Center, NLC «Zhangir Khan West-Kazakhstan Agrarian Technical University», Zhangir Khan Str. 51, Uralsk 090000, Kazakhstan; vadimkst19@gmail.com; 3 Department of Natural Sciences, Kostanay Social-Technical University named after Academician Zulharnai Aldamjar, Kostanay 110000, Kazakhstan; sulejmanovakulaj@gmail.com; 4 Department of Sheep and Goat Breeding, Faculty of Animal Bioengineering, University of Warmia and Mazury in Olsztyn, 10-719 Olsztyn, Poland; micinsk@uwm.edu.pl; 5 Department of Food Security and Biotechnology, NLC «Akhmet Baitursynuly Kostanay Regional University», Kostanay 110000, Kazakhstan

**Keywords:** Aberdeen Angus cattle, adaptation, blood metabolite, microsatellite, genetic information

## Abstract

This article presents the findings of a scientific study investigating the efficacy of various assessment techniques used to evaluate the adaptability and productive qualities of Aberdeen Angus cattle on three prominent farms in the northern region of the Republic of Kazakhstan. A comprehensive analysis of the haematological and biochemical parameters of experimental groups of cattle with different genotypes (American, Canadian, and Estonian selection) was conducted. The studies revealed notable variability in haematological and biochemical indicators, contingent on the origin. Concurrently, the dynamics of the aforementioned indicators did not exceed the physiological norms. The modern allelofund was characterised with the help of microsatellite markers, and the level of genetic diversity of Aberdeen Angus cattle of different genotypes was estimated. The research uncovered the genealogical structure of the populations, the purity of the populations, the provenance, the polymorphism level, the heterozygosity indices, and the Wright fixation index (Fis). The genotyping of cattle of the Aberdeen Angus breed on 15 microsatellite markers yielded the establishment of 80 alleles in the Kolos-firm LLP, 77 alleles in the Vishnevskoe LLP, and 92 alleles in the Sever-Agro N LLP. The total expected heterozygosity was He = 0.673, while the observed heterozygosity was Ho = 0.710.

## Simple Summary

Providing the population with food of animal origin, including beef, is an important task of the agro-industrial complex in the Republic of Kazakhstan. This task can be solved through the effective use of imported Aberdeen Angus cattle. Scientific research in this direction is also relevant for the northern region of the Republic of Kazakhstan. This was where the first comprehensive assessment of the productive traits of imported Aberdeen Angus cattle of different genotypes was conducted, which considered biochemical and haematological blood parameters and molecular genetic characteristics to determine the allelofund of animals via the polymorphism of various genome elements. This enabled a deeper study of the biological basis of the inheritance of productive traits from one generation to the next. Applying such a complex evaluation system will allow us to obtain new results and introduce them into state breeding programmes to develop beef cattle breeding in the future. The breeding of Aberdeen Angus in new conditions in the Republic of Kazakhstan is associated with many factors that affect productivity and depend on animals’ adaptation and acclimatisation processes. Given this connection, it is necessary to apply effective accounting methods to estimate the adaptation and productive qualities of animals.

## 1. Introduction

Northern Kazakhstan is a significant region for livestock production, with vast natural pastures conducive to beef cattle breeding.

Meat cattle breeding represents the primary branch of cattle breeding in Kazakhstan, resulting in the widespread distribution of beef breeds across diverse natural and economic zones and a significant contribution of beef to the total livestock product mass. This encompasses both local breeds, such as Kazakh white-headed, Auliekol, and imported breeds, including Hereford, Aberdeen Angus, and Kalmyk [1,2].

In order to achieve stable development in beef cattle breeding, it is essential to prioritise the acceleration of genetic improvement in breeds and animal types that demonstrate greater productivity [3,4].

One of the most important issues in beef cattle breeding is expanding the area and optimising the breed composition of Aberdeen Angus cattle according to different genotypes of origin. This is because Aberdeen Angus cattle have great potential in terms of meat productivity, good feed returns through weight gain, good enough meat, and young stock fattening. They are distinguished by their uniqueness, namely, the ability to survive in harsh conditions, high productive and reproductive traits, and superior meat productivity compared to other breeds. Furthermore, with improved exploitation, their productivity can be even higher [5,6].

In this regard, since 2011, the Aberdeen Angus breed has been imported into the Republic of Kazakhstan from abroad with the objective of creating breeding reproducers and developing specialised beef cattle breeding in general. However, imported cattle are subjected to several stressful influences, including harsh climatic conditions, a new level of feeding, unbalanced rations in terms of nutrients and biologically active substances, and a lack of active exercise. During the relocation process from one natural climatic zone to another, significant alterations are observed, affecting blood composition, resistance indicators, immune status, productivity, reproductive ability, and numerous other vital parameters. The impact of these unfavourable factors frequently results in the onset of disease and animal mortality. The productivity, safety, and health of animals are directly related to the extent to which specific functions within the animal’s organism are adapted to external conditions [7,8].

The northern region of the Republic of Kazakhstan is home to 79.668 beef cattle, with the Kostanay region accounting for 51.7% (41.208 heads) and the North Kazakhstan region representing 48.2% (38.460 heads).

Aberdeen Angus cattle in the Kostanay region are ranked third in number, representing 22.4% (9.248 heads) of the total, following the domestic breeds of Auliekol cattle (39.1%, 16,116 heads) and Kazakh white-headed cattle (25.9%, 10.685 heads). In the North Kazakhstan region, the Aberdeen Angus breed is ranked second, representing 32.8% (12.634 heads) of the total cattle population. This is followed by the domestic Kazakh white-headed breed, which accounts for 44.4% (17.109 heads). In comparison, an investigation of the North Kazakhstan region revealed 4.2% Aberdeen Angus cattle (3.386 heads) [4].

The programme ‘Kazakhstan—2030’ emphasises the necessity of achieving an increase in livestock productivity and making greater use of opportunities for growing beef production through the intensive growing and fattening of young cattle of various breeds, including Aberdeen Angus, as well as expanding the network of breeding farms [9]. In this regard, a system of measures is required to utilise the achievements of breeding and genetics, as well as new biological methods of determining the economics of herds. This will result in qualitative stabilisation and an increase in the valuable gene pool of Aberdeen Angus cattle [10].

The practice and experience of beef cattle breeding in many foreign countries with developed beef cattle breeding shows that the intensification of this branch requires new, more effective methods of breeding work.

This primarily concerns the broad application of genetic research findings to enhance existing animal varieties and develop new ones that align with contemporary standards. The phenotypic improvement of animals is contingent upon the accurate and reliable evaluation of their genotype, which represents the hereditary basis of the phenotype and productivity [11,12,13,14]. It is essential to consider the distinction between genotype and phenotype, as the ratio between them is unequal. It is the genotype that determines the breeding qualities of animals and their reaction to environmental conditions. Consequently, the same genotype, when subjected to different environmental conditions, results in the emergence of disparate phenotypes.

The primary indicator of the formation of the principal signs of productivity in the animal organism is blood, which is a fundamental system for evaluating the organism. Blood plays a significant role in maintaining not only the organism’s physiological, biochemical, and haematological characteristics but also the quantitative and qualitative indicators of its composition. It responds to the impact of external and internal factors, thereby influencing the organism’s overall health and productivity. Furthermore, it is crucial to ascertain the indicators of natural resistance, which enable the evaluation of an animal’s capacity to adapt and acclimatise to novel conditions. In order to assess the aforementioned capabilities of the animal organism, several blood indicators are typically employed. These indicators enable the natural resistance of the animal organism and its capabilities of adaptation to new conditions of the external environment of feeding and housing to be determined with a high level of reliability [15,16,17].

In order to conduct a scientifically based assessment of the adaptation and productive qualities of Aberdeen Angus cattle, it is of great importance to study the polymorphism peculiarities of the different genome elements. This allows for a deeper insight into the biological basis of the inheritance of productive qualities from parents to offspring.

Recently, the evaluation of genetic resources using biochemical, haematological, and molecular genetic traits has been widely used to confirm the reliability of the origin of breeding animals [18,19,20,21,22]. Microsatellites are employed as DNA markers, facilitating the utilisation of analytical data in cattle breeding [23,24,25,26].

Therefore, when breeding Aberdeen Angus cattle in the northern region of Kazakhstan, it is necessary to study the common origin and unique productive traits that became the basis for the selection of this breed in order to expand the range of Aberdeen Angus cattle in the future.

The aim of this study is to examine the blood clinical status and polymorphism of microsatellite DNA loci in Aberdeen Angus cattle of different genotypes bred in the territory of the northern region of Kazakhstan, specifically in Kostanay and North Kazakhstan regions.

## 2. Materials and Methods

### 2.1. Study Area

The study area is situated in a location that is particularly conducive to agricultural development, with a particular focus on the breeding of beef cattle. A distinctive feature of the region’s climate is its pronounced continentality, characterised by hot and dry summers and cold winters with minimal snowfall.

Northern Kazakhstan, comprising the regions of Kostanay and North Kazakhstan, is an important centre of pedigree beef cattle breeding, symbolising one of the main specialisation zones of Kazakh cattle breeding.

### 2.2. Research Conditions

The research was conducted in 2023–2024 under the conditions of Kolos-firm LLP Sever Agro-N LLP of the Kostanay region and Vishnevskoe LLP of the North Kazakhstan region.

In this study, three groups of cattle of the Aberdeen Angus breed were formed by selection according to different origins and kept together in two farms in the Kostanay region, Kolos-firm LLP and Sever Agro N LLP, and in the North Kazakhstan region, they were kept in Vishnevskoe LLP, which, according to the data in Table 1, represented group I of Estonian cows (n = 34 heads), group II of American cows (n = 42 heads), and group III of Canadian cows (n = 30 heads). The animals were similar in age (4 years old).

In modern beef cattle breeding programmes, the main task is evaluating breeding values. The first step is the distribution of cattle in the herd by class composition, conducted according to the requirements of the current instruction for determining breeding value [27].

The next stage is to study the genealogical structure of the herd in relation to the genotypes studied in order to reveal the principles of identifying animals from the best world genetic resources since this enables the most rational determination and use of the hereditary wealth of the breed, concentrating the most valuable qualities in the best lines and then, as a result of their accelerated development and simultaneous displacement or absorption of the less valuable part of the population, increasing the quality level of the whole breed.

The Aberdeen Angus breeds were maintained under identical feeding and housing conditions. During the research period, the feed for the experimental cattle on the three studied farms was rationed in accordance with the detailed norms of feeding, which considered the physiological needs of the animals [28].

### 2.3. Sample Collection

Blood samples and hair follicles for evaluation were taken from the breeding stock of cows with different genotypes.

To examine haematological and biochemical blood parameters, blood was collected from the jugular vein of experimental animals three to four hours after morning feeding into sterile syringe tubes and 10 mL tubes with ethylene diamine tetra-acetic acid (EDTA) anticoagulant [29,30]. For this study, each group consisted of 15 animals, as recommended by the author [31].

Hair was packaged in blank paper envelopes and clearly labelled for identification. The samples were stored at room temperature. Delivery to the laboratory was performed within 1 month to minimise DNA loss due to follicular drying and shedding [32].

Studies of blood and hair follicles were conducted at the Research Institute of Applied Biotechnology of Akhmet Baitursynuly Kostanay Regional University.

### 2.4. Serum Biochemical Analysis

Biochemical analysis of the centrifuged serum from the Eppendorf MiniSpin (Hamburg, Germany) was conducted on an automatic biochemical analyser, BioChem FC-120 (High Technology Inc., North Attleborough, MA, USA), using HTI Technology reagents. The concentrations of total protein, glucose, and iron, as well as the aminotransferase activities of AST and ALT, were determined.

### 2.5. Haematological Blood Tests

The concentrations of haemoglobin, erythrocytes, leucocytes, and platelets in whole blood were determined using a veterinary haematological analyser, Exigo 17 (Boule Diagnostics AB, Spanga, Sweden).

### 2.6. DNA Extraction

Genomic DNA from hair follicles was isolated using the DNA-Extran-2 kit (LLC ‘SINTOL’, Russia). A negative control tube (‘reagent blank’) containing only DNA extraction reagents without a sample was included in each batch of processed samples to trace the possible occurrence of DNA contamination.

The concentration of double-stranded DNA was measured using a Dynamica Halo DNAmaster (Dynamica, Livingston, UK), and the purity of the DNA solutions was checked by determining the ratio of the absorption at 260 and 280 nm (OD260/280) on a NanoDrop 8000 instrument (Thermo Fisher Scientific Inc., Waltham, MA, USA). Only samples with a >1 ng/µL dsDNA concentration were used for the study.

### 2.7. Microsatellite Genotyping

The samples were genotyped for 15 highly polymorphic microsatellite loci (BM1818, ETH3, CSSM66, INRA23, ILSTS6, TGLA227, TGLA126, TGLA122, SPS115, ETH225, TGLA53, CSRM60, BM2113, BM1824, and ETH10) recommended by the International Society of Animal Genetics (ISAG) [33]. The multiplex PCRs were used in a final volume of 10 µL in a PCR buffer containing 200 mM dNTPs, 1.0 mM MgCl2, a 0.5 mM primer mix, 1 unit of Taq polymerase (LLC ‘Gordiz’, Moscow, Russia), and 1 µL of genomic DNA. Initial denaturation at 95 °C for 4 min was followed by 35 cycles of PCR amplification (95 °C, 20 s; 63 °C, 30 s; 72 °C, 1 min). A final extension was performed at 72 °C for 10 min. Negative controls (PCR reaction without DNA template) were included in each PCR experiment to check for possible DNA contamination. Amplification reactions were performed according to the thermocycler genotyping kit manufacturer’s instructions (Applied Biosystems, Beverly, MA, USA). Fragment length determination was performed on an automated AB 3500 Genetic Analyzer (Applied Biosystems, Beverly, MA, USA) according to the manufacturer’s instructions. The interpretation of analysis results was performed using Gene Mapper 6.0 software (Applied Biosystems, Beverly, MA, USA). Allele sizes were standardised according to an ISAG International Bovine (Bos Taurus) STR Typing Comparison Test (2018–2019).

### 2.8. Data Analysis

The indices of observed heterozygosity (Ho), expected heterozygosity (He), and coefficient of inbreeding (FIS) were calculated using Genalex 6.5 [34]. Private alleles for any population were considered alleles unique for a population and detected in at least 25% of the sample of the population. Data were crosschecked using FSTAT and Genepop.

Haematological and biochemical blood data are presented as the mean ± standard deviation (X ± Sx). The main numerical material obtained during the research was processed using variation statistics with the software package SPSS Base 22 for Windows [35].

## 3. Results

### 3.1. Distribution of Aberdeen Angus Cattle of Different Genotypes by Breeding Value

The significance of purebred breeding in the context of beef cattle breeding is considerable, as it exerts a pivotal influence on the expansion of the population with valuable breeding traits. This is particularly evident in the selection and pedigree work pertaining to the breed.

Table 2 presents the statistics of the cattle population of the studied farms in terms of number and distribution according to the grading class for the study period. The data indicate a tendency towards an increase in the absolute number of animals, both within the herd as a whole and across the various classes.

A detailed examination of the numerical data presented in Table 2 reveals a striking concentration of Aberdeen Angus cattle within the ‘elite record’ category across all studied farms. In the Kolos-firm LLP, 41% of the livestock is classified as belonging to the ‘elite record’ class, while in the Vishnevskoe LLP, this figure is lower (36.5%). The lowest observed indicator is 35%, recorded in the Sever Agro N LLP.

The greatest specific weight is occupied by cows of factory lines ABAJA PORTOS 95283 (39.8%) and ABAJA ELVIS 9528 (34.9%), as well as Connealy Skipper and FRANKIE 95259 (3%) in LLP Kolos firm. Furthermore, in Vishnevskoe LLP, approximately 10% of the cows belong to the S7RBARRISTER 45X line, while a slightly smaller proportion (7.6%) are of the BH BRUIN 54X line and the domestic Henri KZT157789649 line.

### 3.2. Genealogical Structure of the Herd

In planning breeding work, it is logical to adhere to the breeding programme of the Aberdeen Angus breed, which is designed to preserve essential traits in the breed within the context of the northern region of Kazakhstan. This depends on the inheritance and expression of traits in a complex manner.

To further enhance the productive qualities of the Aberdeen Angus breed on the farms of Sever-Agro N LLP, Kolos-firm LLP, and Vishnevskoe LLP, it is of paramount importance to select an appropriate breeding strategy and methodology, as well as suitable selection criteria, which should be informed by an understanding of the genealogical structure.

Analyses of changes in the structure of pedigrees reveal the nature of the direction of selection of the best genotypes of cows and proven bulls, which ensures the production of high-quality animals. Consequently, it is essential to ascertain whether there is a narrowing of the gene pool in these populations, as this could have a detrimental impact on the viability and productive qualities of the animals. To address this issue, it is essential to implement a rigorous and continuous monitoring programme to support the genetic use of the breed [36,37,38,39,40]. This entails a set of measures designed to regulate and enhance the breeding and improvement of animals through the analysis of population structure and the determination of allele and genotype frequencies.

In this context, the subsequent phase of the study focused on the genealogical structure of the Aberdeen Angus cattle herd to elucidate the principles underlying the formation of the breeding stock hierarchy and determine strategies for future breed improvement. This approach allows for the most rational determination and utilisation of the breed’s hereditary wealth. By concentrating the most valuable genotypes from the best bulls, which are producers of imported breeding, and as a result of their accelerated analysis, the most valuable genotypes can be selected from the best bulls, which, in turn, results in an improvement in the breed.

The development of beef cattle breeding in the studied farms, namely, Kolos-firm LLP, Sever-Agro N LLP, and Vishnevskoe LLP, is undergoing a period of significant advancement. This is evidenced by the implementation of a comprehensive selection programme for cattle of the Aberdeen Angus breed, the establishment of a dedicated breeding nucleus, and the introduction of new genetic material from foreign countries, which has contributed to the evolution of the modern breeding stock observed in the studied farms. The specific weight of the total herd on the farms is as follows: in Kolos-firm LLP, the Kazakhstan selection accounts for 74.2%, while the Estonian selection accounts for 25.5%. In Sever-Agro-N LLP, the herd comprises 85.5% American and 14.5% Kazakh selections. In Vishnevskoe LLP, the cows are of Canadian (55.7%) and Kazakh selection (44.3%).

The realisation of genetic potential in the breed of pedigree animals can be traced in the context of the studied farms according to the data in Table 3. This allows us to determine the rational use of some outstanding genotypes, individual animals, and their groups for herd improvement.

The genealogical structure of the breeding herd in the studied farms is represented by a list of bulls (producers), the largest number of offspring of which can be seen in the analysis of Table 3 in Kolos-firm LLP from EE 16966079 ABAJA PATRICK 95305 (11.7% daughters of cows (39 heads)) and bull DK 1989501341 FREDERIK 95220 (10.5% (35 heads)).

In Vishnevskoe LLP, the greatest number of daughters of cows was established for S7R BARRISTER 45X 1639080 and JL DISTRICT 0311 159405 bulls, at a rate of 10% for each (13 heads). In Sever-Agro N LLP, the greatest number of cows were derived from the FREYS MULBERRY 507A 1630287 bull, representing 0.44% of the total (8 heads).

### 3.3. A Comparative Analysis of the Haematological and Biochemical Blood Parameters of Experimental Groups of Cattle of Different Genotypes

Haematological parameters, including erythrocyte, leucocyte, and platelet counts, did not differ between groups and were within normal limits (Table 4).

The analysis was carried out in a comparative aspect for some blood parameters. The content of the parameter “haemoglobin” in the blood showed the advantage of Canadian breeding cows of group III at the age of 4 years by 3% and 2.7% (3.25 and 3 g/L) compared to group I cows of Estonian breeding and group II cows of American breeding. A similar trend was observed at the age of five years. This suggests that the animals in group III have a high metabolic rate at four and five years old, which corroborates the observation of high productivity in animals in the northern region of Kazakhstan.

The intensity of protein metabolism can be estimated to a certain extent by examining the biological composition of the blood. Blood serum proteins constitute the material from which proteins of all organs and tissues are formed. The concentration of substances in the blood serum allows for the estimation of the intensity of metabolic processes occurring in the bodies of animals. This indicator is susceptible to alteration by external and internal factors [29]. Significant differences were observed in the protein content of the experimental animals. The total protein concentration in the blood serum of cows exhibited a significant advantage in Canadian cows four years old, with a difference of 8.3% and 4.9% (6.79 and 4 g/L) compared to the coevals of Estonian and American cows. It is also noteworthy that the superiority at the age of five is similarly reflected in the data.

The assessment of the state of carbohydrate metabolism in the animal organism and the determination of its role as the primary energy source in the body is achieved through the measurement of the ‘glucose’ parameter [41,42]. The glucose concentration in the blood was significantly higher in four-year-old American cows from group I, with a mean value of 1.07 mmol/L, representing a 32.1% increase over the mean value of 0.06 mmol/L observed in Estonian and Canadian cows. Similarly, at five years old, the superiority of the American selection was observed compared to the Estonian and Canadian selections.

The mineral supply of metabolic processes within the body is essential for animals’ normal growth and development. Mineral substances, including iron and potassium, are integral components of body cells and are pivotal in regulating physiological processes. They serve as crucial indicators of mineral metabolism. The concentration of iron in serum depends upon iron absorption in the intestine, the degree of decay or loss of haemoglobin, and the amount of haemoglobin biosynthesis. Biochemical analysis revealed that the iron concentration was within the normal range.

The primary components of protein metabolism in the animal organism are the processes of overamination, which are conducted by aspartate transaminotransferase (ACT) and alanine transaminotransferase (ALT), and the reverse transfer of the amine group of amino acids to keto acids. The analysis of transaminase activity, as illustrated in Table 1, revealed that cows of varying ages in the American selection exhibited a modest increase in aspartate transaminase activity at four years old compared to Estonian (17.4%, 14.4 U/L) and Canadian cows (14.5%, 12.04 U/L). The investigation into alanine aminotransferase activity revealed no notable discrepancies in this parameter between animals of diverse breeds.

The numerous reactions of various systems and organs that an animal organism employs to adapt to different influences (such as housing and feeding) are characterised by the activity of alkaline phosphatase, which regulates the active transport of phosphorus and calcium and, in general, the ‘bone–tissue–blood’ system. The biochemical indicator, namely, the concentration of alkaline phosphatase, was within the physiological norm in the studied groups of cows of different breeds, which indicates that there was an increase in the control of balanced animal feeding on the farm [43,44].

### 3.4. Microsatellite Analysis of Aberdeen Angus Cattle of Different Genotypes

Despite the widespread distribution of the Aberdeen Angus cattle breed, its genetic structure remains insufficiently studied. To address this knowledge gap, we conducted a microsatellite DNA analysis, presented in Table 5, to acquire further insights into the genetic diversity of the studied cattle herds. This analysis will inform the breeding and pedigree work on each farm, as outlined in Table 1.

**Figure 1:**
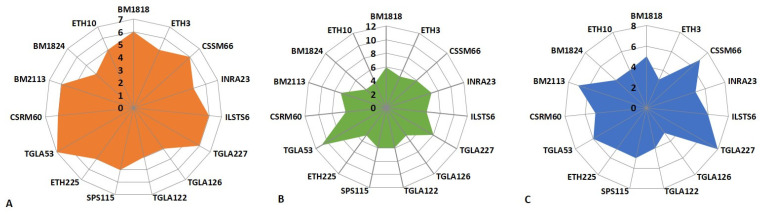
The dynamics of the allele number in the microsatellite loci investigated in cattle: (**A**)—Estonian selection in Kolos-firm LLP; (**B**)—American selection in Sever-Agro N LLP; (**C**)—Canadian selection in Vishnevskoe LLP.

As illustrated in Table 5 and Figure 1, the analysis of the obtained results revealed that the studied samples of Aberdeen Angus cattle exhibited polymorphism at all microsatellite loci. The genotyping of cattle of the Aberdeen Angus breed on 15 panel STR loci revealed the presence of 80 alleles in Kolos-Firm LLP, 77 in Vishnevskoe LLP, and 92 in Sever-Agro N LLP. The number of alleles observed at the loci exhibited considerable variation, with values ranging from four (TGLA126, TGLA122, and BM1824) to seven (TGLA53) in the Kolos-Firm LLP, from three (ETH3) to eight (TGLA227) in the Vishnevskoye LLP, and from four (BM1824 and ETH10) to eleven (TGLA53) in the Sever-Agro N LLP.

The locus TGLA53 exhibited the greatest variability in the Estonian cattle selection of the Kolos-firm LLP, with allele 7 (3.769) displaying the highest polymorphism level. The second position is occupied by loci with allele 6, namely, BM1818 (polymorphism level: 2.521), CSSM66 (polymorphism level: 4.368), ILSTS6 (polymorphism level: 4.667), TGLA227 (polymorphism level: 4.404), CSRM60 (polymorphism level: 2.846), and BM2113 (polymorphism level: 4.417).

In the Vishnevskoe LLP, the locus TGLA227 exhibited superior performance in the Canadian cattle selection, with allele 8 (4.455) displaying the highest polymorphism level. The loci with allele 7, namely CSSM66 (polymorphism level—3.722) and BM2113 (polymorphism level—4.873), were ranked second.

In Sever-Agro N LLP, the locus TGLA53 was found to be superior, with allele 11 (3.948) exhibiting the highest polymorphism level. The locus with allele 8, TGLA227 (polymorphism level—4.900), and the locus with allele 7, INRA23 (polymorphism level—2.210), were the second-best performers.

Furthermore, we defined the parameter ‘Wright fixation index (Fis)’, which reveals deviations of heterozygous genotypes from the theoretically expected ones. The Wright fixation index (Fis) can assume both positive and negative values, which, in turn, distinguish between deficiencies and excesses of heterozygotes. A negative value of the Wright fixation index (Fis) indicates an excess of heterozygosity resulting from negative haphazard crossing or the use of heterozygotes as a selection criterion. Conversely, a positive value indicates a deficiency of heterozygous individuals, while a value of 0 signifies random mating [45,46,47].

The results of the analysis, based on the data presented in Table 5, revealed that Wright’s fixation index (Fis) indicated a lack of heterozygotes in the Estonian cattle selection of the Kolos-firm LLP at six loci. The results demonstrated that ETH3 (0.022), INRA23 (0.061), ILSTS6 (0.045), TGLA126 (0.184), SPS115 (0.085), and TGLA53 (0.076) exhibited a fixation index that was not statistically significant, with values ranging from 0.022 to 0.184. An excess of heterozygotes was observed at nine loci, with the greatest excess observed at BM1818, CSSM66, TGLA227, TGLA122, ETH225, CSRM60, BM2113, BM1824, and ETH10, ranging from −0.003 at BM1824 to −0.204 at CSSM66. The mean fixation index for the 15 loci was −0.499, indicating an excess of heterozygotes.

The data from the genetic structure study were summarised, and the resulting values of the effective allele number, heterozygosity, and Wright’s fixation level (Wright) were calculated. The data are presented in Table 6.

This table includes indicates of the effective number of alleles (Ne), observed (Ho) and expected (He) heterozygosity values, and inbreeding coefficients (Fis) of the investigated herds.

The observed number of alleles at the 15 loci studied ranged from 4 to 11. The mean effective number of alleles was 3.287. The total expected heterozygosity was 0.673, and the observed heterozygosity was 0.710 (Table 6). Heterozygosity on the farms studied ranged from 0.695 to 0.727, in agreement with the above values.

## 4. Discussion

In the context of the current conditions in the Republic of Kazakhstan, the optimal strategy for preserving and enhancing the imported gene pool of Aberdeen Angus cattle with diverse genotypes (including American, Canadian, and Estonian selections) hinges on the advancement of breeding methodologies that are grounded in the latest insights from population genetics.

It is of the utmost importance to ensure the accuracy and objectivity of evaluating breeding qualities, which are genetically determined in animals. A comprehensive examination of the principal productive characteristics of beef cattle in the context of the northern region of Kazakhstan is a valuable undertaking.

In this regard, the choice of effective evaluation methods will play a significant role, including both breeding strategy and tactics. This allows for improvements to be made to the methods of selection and breeding work to increase meat productivity and improve quality in order to meet the needs of the population of Kazakhstan.

The conducted scientific research demonstrated positive dynamics in the choice of estimation methods of the adaptation and productive qualities of imported Aberdeen Angus cattle with different genotypes.

The biochemical composition of the blood serum of Aberdeen Angus cattle of different genotypes in different regions in northern Kazakhstan was within the normal range under identical conditions of keeping and feeding.

The results demonstrate that Aberdeen Angus cattle imported from diverse foreign countries exhibit robust adaptive capabilities, which bodes well for their breeding in the northern region of Kazakhstan, where climatic conditions are similar.

As evidenced in Table 5, the Wright fixation index (Fis) analysis of the Canadian selection cattle at Vishnevskoe LLP revealed a dearth of heterozygotes at five loci. The following loci exhibited a lack of heterozygotes: CSSM66, SPS115, ETH225, CSRM60, and BM1824. Similarly, the Wright fixation index (Fis) was also insignificant in magnitude, with values ranging from 0.081 (locus CSRM60) to 0.267 (locus SPS115). An excess of heterozygotes was observed at ten loci: BM1818, ETH3, INRA23, ILSTS6, TGLA227, TGLA126, TGLA122, TGLA53, BM2113, and ETH10. The fixation index for Wright ranged from −0.016 at the TGLA122 locus to −0.293 at the ETH10 locus. The mean fixation index across the 15 loci was −0.042, which also indicated an excess of heterozygotes.

In LLP Sever-Agro N, the data analysis in Table 5 revealed that the parameter ‘Wright’s fixation index (Fis)’ was similar to that observed in the Kolos-firm LLP and Vishnevskoe LLP peers, which indicated a lack of heterozygotes at three loci: ILSTS6, TGLA122, and SPS115. The magnitude of Wright’s fixation index (Fis) was also insignificant, with values ranging from 0.008 (at the ILSTS6 locus) to 0.036 (at the TGLA122 locus). An excess of heterozygotes was observed at twelve loci: BM1818, ETH3, INRA23, TGLA227, TGLA126, CSSM66, TGLA53, BM2113, ETH225, CSRM60, BM1824, and ETH10. The Wright fixation index (Fis) ranged from −0.024 at the BM2113 locus to −0.355 at the ETH3 locus. The mean fixation index across the 15 loci was −1.4, indicating an excess of heterozygotes in the herd of this enterprise.

In accordance with Table 5, a review of the results reveals that the ‘Level of polymorphism’ is the primary indicator in the studied experimental groups of cattle in Kolos-firm LLP, Vishnevskoe LLP, and Sever-Agro N LLP. This parameter effectively reveals the alleles operating in the populations and correlates with the number of alleles detected in the studied loci.

The most prevalent alleles among Aberdeen Angus cattle of diverse genotypes were alleles 6 and 8 of the TGLA227 locus, identified in 8.8% of cases (Vishnevskoye LLP and Sever-Agro N LLP). Alleles 7 and 11 of the TGLA53 locus were identified in 9.6% of cases by Kolos-Firma LLP and Sever-Agro N LLP. Alleles 6 and 7 of the CSSM66 locus were identified in 7.6% of cases, as observed in the Kolos-Firm LLP, Vishnevskoye LLP, and Sever-Agro N LLP samples. Allele 7 at the BM2113 locus was identified in 8.0% of cases in the Vishnevskoye and Sever-Agro N LLPs. Therefore, it can be concluded that an increase in the number of alleles detected corresponds to an elevated level of polymorphism.

The utilisation of microsatellite analysis to regulate microevolutionary processes in herds of Aberdeen Angus cattle in the northern region of Kazakhstan permitted the investigation of the genetic resources of the breed, the determination of its genetic structure, and the monitoring of said structure in order to prevent a decrease in genetic diversity. Furthermore, the analysis provided material for developing long-term breeding programmes designed to enhance the breed in the future.

Our results are consistent with similar studies by leading authors. For example, in the Argentine Angus herd, the observed heterozygosity using nine microsatellites (n = 59) was 0.715 [48]. In British Angus, the observed heterozygosity using twelve microsatellites (n = 33) was 0.428 [49]. Twelve microsatellites and 1369 Angus individuals bred in Hungary gave a Ho of 0.710 [37]. Seven microsatellites and 52 Angus individuals bred in Brazil gave a Ho of 0.491 [50]. Twenty-two microsatellites on 164 Canadian Angus [51] gave a Ho of 0.630. The Ho values for old- and new-type Colombian Angus (n = 29) were 0.734 and 0.707, respectively [52].

High values of the parameters of observed and expected heterozygosity indicate the use of an open breeding system for Aberdeen Angus cattle of different genotypes in the farms studied and a low level of inbreeding in these herds. This is consistent with the conclusions of many leading scientists that a low frequency (He) in the sample is characteristic of a closed breeding system, and a high frequency is characteristic of an open breeding system [53,54].

## 5. Conclusions

This research work showed that the simultaneous use of several approaches to assess the breeding value of Aberdeen Angus cattle is effective.

A positive trend of increasing the number of Aberdeen Angus cattle in the studied herds was observed according to the distribution by breeding value classes. The highest class (‘elite record’) comprised 41% of the livestock in Kolos-firm LLP, 36.5% in Vishnevskoye LLP, and 35% in Sever Agro-N LLP.

The genealogical structure of the modern breeding stock in the studied farms has different genotypes of local Kazakh and different genotypes of imported selection. Thus, the Kazakh and Estonian selections comprised 74.2% and 25.5% of the total livestock in Kolos-firma LLP, respectively. In Sever-Agro-N LLP, Kazakh livestock comprised 14.5%, and American livestock comprised 85.5%. In Vishnevskoye LLP, Kazakh cows comprised 44.3%, and Canadian cows comprised 55.7%.

The study of haematological and biochemical indices of blood and the content of formed elements of blood did not reveal any sharp differences between the studied groups of animals of different genotypes. This indicates the high adaptability of animals of different genotypes to the environmental conditions of the northern region of the Republic of Kazakhstan, which is a significant indicator for ensuring the safety of young animals, improving their productive qualities, and increasing the number of livestock.

The experimental groups of livestock with different genotypes in Kolos-firm LLP, Sever Agro N LLP, and Vishnevskoe LLP were genotyped for 15 microsatellite loci, which are appropriate for characterising the allele frequency of the studied breed. The resulting genetic structure of the populations demonstrated a high level of polymorphism and codominant inheritance of the studied loci.

The identified characteristics of STR polymorphism in the studied Aberdeen Angus cattle populations with varying genotypes enable genetic monitoring and the maintenance of genetic diversity within gene pool herds, as well as more effective utilisation of the aforementioned loci in genetic population studies.

A positive trend was identified for the further use of the best-proven breeding Estonian, Canadian, and American bulls in reproduction.

## Figures and Tables

**Table 1:** The formation of experimental groups of Aberdeen Angus cattle is contingent upon the specific genotypes and heads involved.

Group	Place of Selection	Genotype	n
I	Kolos-Firm LLP	Estonian	34
II	Sever Agro-N LLP	American	42
III	Vishnevskoe LLP	Canadian	30
	Total		106

n—the number of animals.

**Table 2:** The distribution of Aberdeen Angus cattle by breeding value (class).

Indicator	North-Agro N LLP				Kolos-Firm LLP				Vishnevskoye LLP			
	Classified by Class, %											
	By Herd		By Cows		By Herd		By Cows		By Herd		By Cows	
	Heads	%	Heads	%	Heads	%	Heads	%	Heads	%	Heads	%
Elite Record	964	35	850	47.4	362	41	77	23.2	103	36.5	45	34.6
Elite	1548	56.2	782	43.6	148	18.1	29	8,7	143	50.7	70	53.8
1st grade	243	8.8	163	9	255	13.2	15	4.5	36	12.7	15	11.6
Total	2755	100	1795	100	836	100	331	100	282	100	130	100

**Table 3:** The genealogical structure of the breeding herd by affiliation from imported breeding bulls.

Breeding Bulls Used on the Farm, Nickname, Individual Number	Total Number of Cows per Bull in the Herd
Kolos-firm LLP	
DK 1989501341 FREDERIK 95220	35
EBA Eagle Bando 1114 KZP156547172	24
EE 14465116 ABAJA PORTOS 95283	25
EE 16966079 ABAJA PATRICK 95305	39
Other lines	208
Vishnevskoye LLP	
BH BRUIN 54X 1644270	10
S7R BARRISTER 45X 1639080	13
JL DISTRICT 0311 1594050	13
Other lines	94
North-Agro N LLP	
FREYS MULBERRY 507A 1630287	8
1710517 OKCC TRADIN LEGEND 228B	6
1710509 OKCC KING OF TRADE 264B	6
3998210 F A S R GRADE UP 4091	6
3998220 WILD WIND 4GM4	6
3880703 ONION HOLLOW CAPITALIST 527	6
Other lines	1757

**Table 4:** The haematological and biochemical composition of cow blood (X ± Sx).

Indicators	Norm	Group					
		I		II		III	
		Cows 4 Years Old	Cows 5 Years or Older	Cows 4 Years Old	Cows 5 Years or Older	Cows 4 Years Old	Cows 5 Years or Older
n		15	15	15	15	15	15
Erythrocytes, 10^12^/L	5.0–10.0	5.88 ± 0.13	5.81 ± 0.22	5.91 ± 0.16	5.86 ± 0.17	5.86 ± 0.18	5.84 ± 0.25
Leucocytes, 10^9^/L	4.0–12.0	8.15 ± 0.28	8.53 ± 0.31	7.68 ± 0.37	7.83 ± 0.34	7.85 ± 0.43	8.34 ± 0.13
Platelets, 10^9^/L	100–800	260.1 ± 12.22	259.6 ± 12.99	275 ± 19.51	265.75 ± 16.08	272.5 ± 18.65	270.6 ± 17.02
Haemoglobin, g/L	80–150	108.25 ± 2.43	107.88 ± 1.46	108.5 ± 1.57	110.75 ± 1.98	111.5 ± 2.20	109.8 ± 2.15
Total protein, g/L	72–86	75.31 ± 2.87	75.77 ± 2.86	78.1 ± 2.6	80.6 ± 2.52	82.1 ± 3.2	82.0 ± 2.65
Glucose, mol/L	2.2–3.3	3.34 ± 0.15	3.2 ± 0.19	2.27 ± 0.06	2.52 ± 0.19	3.28 ± 0.28	2.80 ± 0.22
Iron, µmol/L	12.0–42.0	20.57 ± 1.82	18.74 ± 1.96	25.24 ± 2.37	18.9 ± 1.54	22.21 ± 2.62	22.3 ± 1.77
Potassium, µmol/L	4.0–5.8	4.36 ± 0.22	4.29 ± 0.21	4.2 ± 0.20	3.89 ± 0.12	4.40 ± 0.31	4.35 ± 0.55
AST, U/L	46–110	68.76 ± 6.34	69.38 ± 4.90	71.16 ± 4.69	75.06 ± 4.96	83.2 ± 4.55	78.5 ± 4.82
ALT, U/L	6.9–35	32.5 ± 2.99	31.93 ± 2.85	31.98 ± 2.97	33.95 ± 4.27	30.5 ± 2.22	32.62 ± 4.21
Alkaline phosphatase, U/L	18–153	57.8 ± 14.26	55.08 ± 12.28	58.08 ± 12.19	59.13 ± 12.78	57.5 ± 11.85	58.1 ± 12.32

**Table 5:** The values of the main genetic diversity indicators for microsatellite loci of different genotypes of Aberdeen Angus cattle.

Microsatellite Locus	Na	Ae	Ho	He	Fis
Kolos-firm LLP (Estonian selection)					
BM1818	6	2.521	0.607	0.603	−0.006
ETH3	5	2.405	0.571	0.584	0.022
CSSM66	6	2.769	0.929	0.771	−0.204
INRA23	5	2.830	0.607	0.647	0.061
ILSTS6	6	4.667	0.750	0.786	0.045
TGLA227	6	4.404	0.821	0.773	−0.063
TGLA126	4	2.583	0.500	0.613	0.184
TGLA122	4	2.644	0.714	0.622	−0.149
SPS115	5	2.412	0.536	0.585	0.085
ETH225	5	4.010	0.857	0.751	−0.142
TGLA53	7	3.769	0.679	0.735	0.076
CSRM60	6	2.846	0.679	0.649	−0.046
BM2113	6	4.417	0.857	0.774	−0.108
BM1824	4	2.785	0.643	0.641	−0.003
ETH10	5	3.853	0.893	0.740	−0.206
mean (X ± Sx)	5.3 ± 0.23	3.3 ± 0.21	0.7 ± 0.04	0.7 ± 0.02	−0.499
Vishnevskoye LLP (Canadian selection)					
BM1818	5	3.769	0.857	0.735	−0.167
ETH3	3	2.673	0.810	0.626	−0.293
CSSM66	7	3.722	0.667	0.731	0.088
INRA23	5	2.731	0.762	0.634	−0.202
ILSTS6	6	3.528	0.762	0.717	−0.063
TGLA227	8	4.455	0.810	0.776	−0.044
TGLA126	3	2.403	0.714	0.584	−0.223
TGLA122	4	2.285	0.571	0.562	−0.016
SPS115	5	3.500	0.524	0.714	0.267
ETH225	5	3.354	0.619	0.702	0.118
TGLA53	6	4.200	0.952	0.762	−0.250
CSRM60	5	3.645	0.667	0.726	0.081
BM2113	7	4.873	0.857	0.795	−0.078
BM1824	4	2.873	0.571	0.652	0.123
ETH10	4	3.600	0.762	0.722	−0.055
mean (X ± Sx)	5.1 ± 0.38	3.4 ± 0.19	0.7 ± 0.03	0.7 ± 0.02	−0.042
Sever-Agro N LLP (American selection)					
BM1818	6	3.661	0.772	0.727	−0.062
ETH3	5	2.555	0.825	0.609	−0.355
CSSM66	6	4.329	0.772	0.769	−0.004
INRA23	7	2.210	0.667	0.548	−0.218
ILSTS6	6	3.638	0.719	0.725	0.008
TGLA227	8	4.900	0.842	0.796	−0.058
TGLA126	5	2.401	0.632	0.584	−0.082
TGLA122	6	1.572	0.351	0.364	0.036
SPS115	6	2.713	0.614	0.631	0.028
ETH225	5	2.891	0.719	0.654	−0.100
TGLA53	11	3.948	0.789	0.747	−0.057
CSRM60	6	2.723	0.702	0.633	−0.109
BM2113	7	4.059	0.772	0.754	−0.024
BM1824	4	2.249	0.667	0.555	−0.200
ETH10	4	1.922	0.579	0.480	−0.207
mean (X ± Sx)	5.1 ± 0.45	3.1 ± 0.25	0.7 ± 0.03	0.6 ± 0.03	−1.4

Na—the number of alleles per locus; Ae—the level of polymorphism; Ho—observed heterozygosity; He—expected heterozygosity; Fis—Wright’s fixation index.

**Table 6:** The mean values of the genetic diversity indices of the animal samples studied.

Herd Code	Ne	Ho	He	Fis
Kolos-firm LLP	3.368	0.710	0.685	−0.030
Vishnevskoye LLP	3.441	0.727	0.696	−0.048
Sever-Agro N LLP	3.051	0.695	0.638	−0.094
Total	3.287	0.710	0.673	−0.057

## Data Availability

All relevant data are presented within the paper.
